# Predictors of Post-induction Hypotension for Patients With Pulmonary Hypertension

**DOI:** 10.7759/cureus.31887

**Published:** 2022-11-25

**Authors:** Adriano Bellotti, Simrat Arora, Chelsea Gustafson, Ian Funk, Craig Grossheusch, Carter Simmers, Quefeng Li, Yutong Liu, Alan Smeltz

**Affiliations:** 1 Department of Anesthesiology, University of North Carolina, Chapel Hill, USA; 2 Department of Biostatistics, University of North Carolina, Chapel Hill, USA

**Keywords:** surgical risk factors, mean arterial pressure, anesthesia, pulmonary hypertension, post-induction hypotension

## Abstract

Purpose

The purpose is to identify predictors of post-induction hypotension (PIH) during general anesthesia in a population of patients with varying degrees of pulmonary hypertension (PH).

Methods

This is a single-center, retrospective, observational study of perioperative data obtained via electronic health records from patients with PH undergoing surgery over a five-year period. Baseline patient characteristics, peri-induction management variables, and pre-induction mean arterial pressure (MAP) were statistically analyzed using Kruskal-Wallis rank sum tests, Pearson’s chi-squared tests, and logistic regression analysis to identify risk factors for PIH. We further assessed the relationship between PH and PIH using propensity score matching. Primary outcomes include a percent decrease in post-induction blood pressure as well as a post-induction nadir with a threshold of 55 mm Hg.

Results

Eight hundred fifty-seven patients in the cohort stratified by severity of PH reveal that advanced age (p < 0.001), higher BMI (P = 0.002), higher American Society of Anesthesiologists (ASA) score (P = 0.001), and renal and cardiac comorbidities (P < 0.001) are associated with PH severity. None of our tested parameters were significantly predictive for PIH in patients with PH. Right heart failure was found to be weakly and non-significantly predictive of PIH in patients with PH (P = 0.052, odds ratio [OR] = 1.116). Diabetes (P = 0.007, OR = 0.919) and maintenance of spontaneous ventilation (P = 0.012, OR = 0.925) were associated with decreased rates of PIH.

Conclusion

Hypotension after induction of general anesthesia in patients with PH is a serious problem, yet statistically significant risk factors were not identified. History of diabetes and preservation of spontaneous ventilation had a significant but weak effect of decreasing rates of PIH. This pilot study was limited by retrospective design and warrants further analysis with a prospective cohort.

## Introduction

Pulmonary hypertension (PH) is the state of increased blood pressure in the pulmonary vasculature. PH has numerous etiologies as categorized by the World Health Organization (WHO), including primary arterial disease, left heart disease, lung disease, and thromboembolism, among others [1,2]. Population studies suggest the prevalence of PH ranges up to 2.6%, and PH is often comorbid with other anesthetic risk factors, including high body mass index (BMI), left ventricular dysfunction, and systemic hypertension [3]. PH results in several pathophysiologic changes, such as right heart failure and decreased left ventricular preload. In the early stages of PH, right ventricular contractility increases as a homeostatic adaptation to increased pulmonary pressures. When this adaptation fails, these hemodynamic changes can result in reduced stroke volume and low systolic blood pressure [4,5]. Prior studies analyzing 17 million patients in the postoperative period found patients with PH were 43% more likely to suffer adverse cardiac events [6]. In a case-matching study, 62 patients with severe PH had increased rates of delayed extubation and post-operative heart failure leading to increased mortality [7]. In aggregate, these studies among others suggest that patients with all subtypes of PH have increased perioperative morbidity and mortality [8]. Our understanding of these detrimental surgical outcomes in patients with PH is incomplete. Therefore, we aimed to study this association from an intraoperative perspective.

Systemic arterial hypotension after the induction of general anesthesia, henceforth referred to as post-induction hypotension (PIH), is a frequent complication in the operating room [9]. In a study of 4,096 patients, PIH was found to occur occurs in up to 7.7% patients with of American Society of Anesthesiologists (ASA) score I-II and 12.6% with ASA score III-IV [10]. Patients experiencing PIH have a higher incidence of several adverse clinical outcomes from poor perfusion of end-organ systems, including myocardial injury, stroke, and acute kidney injury [11,12]. It is therefore crucial to monitor for PIH during peri-operative management. PIH is of particular interest in the study of patients with hemodynamic changes associated with severe PH. Prior observational studies have identified numerous risk factors for PIH using traditional statistical methods [10,13] and machine learning [14,15]. Thus far, no studies have specifically analyzed PIH in patients with established PH.

In this study, we identified predictors of PIH in a patient population with PH. We hypothesized that the altered hemodynamic sequelae of PH (decompensated right ventricle and decreased left ventricular preload) would predict poor homeostatic adaptation to general anesthesia and thus greater PIH. Decreased left ventricular preload predisposes to decreased systemic blood pressure, and during general anesthesia, which typically further decreases systemic pressures, a hemodynamically compromised circulatory system might result in PIH. This retrospective study analyzes the relationship between preoperative patient parameters such as PH severity with PIH as a marker of intraoperative perfusion.

## Materials and methods

This retrospective observational study was conducted at an academic tertiary care center in the southeastern United States. This study was approved by the Institutional Review Board (Office of Human Research Ethics, IRB# 21-0412) on June 7, 2021.

Data collection

Patient records in the electronic health record system were extracted using BusinessObjects™ (SAP®, Paris, France) software or manual extraction. Sensitive data was stored using REDCap® (Nashville, TN) database software. Second- and third-party data collection teams performed numerous audits for internal quality control to minimize bias and human error. Among the data collected were baseline patient demographics, past medical history, and intraoperative data including vital signs, ASA score, vascular access, and medications administered.

Study protocol and patient population

**Figure 1:**
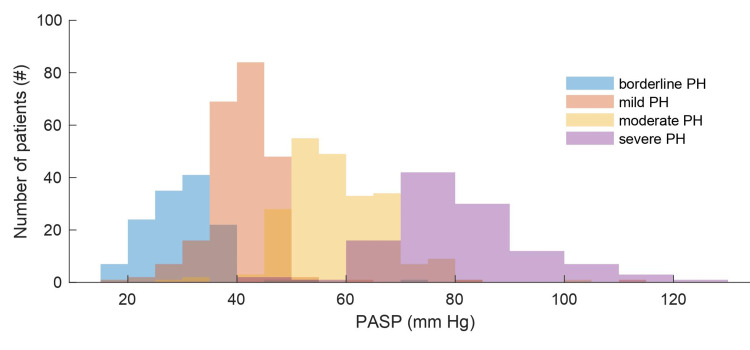
Severity of pulmonary hypertension (PH) delineated by pulmonary artery systolic pressure (PASP) Patients included in this study are discretized according to the severity of PH based on a combination of PASP, right ventricular function and hypertrophy, yet the measured PASP alone provides similar delineation between categories with minimal overlap (indicated by the brown regions).

Patients over 18 years old who underwent any surgery requiring general anesthesia with intubation between October 2015 and December 2020 with PH were included. The diagnosis of PH was determined with the associated International Classification of Disease diagnostic code, ICD-10 I27. PH was diagnosed preoperatively via an echocardiogram. The echocardiogram reports also stratified patients by severity of PH into categories borderline, mild, moderate, severe, and unknown, as estimated by a holistic assessment. This holistic assessment considers clinical findings; past medical history, such as left heart disease or chronic lung disease; and also factors in the estimated pulmonary artery systolic pressure (PASP) in the setting of right ventricular function and degree of hypertrophy. Although there are no definitive grading criteria used by echocardiologists to assign PH severity, this holistic assessment yields a scale strongly associated with PASP, as demonstrated in Figure 1. Patients who were already under general anesthesia or deep sedation prior to entering the operating room were excluded. We also excluded records of patients on mechanical circulatory support. Lastly, incomplete medical records, such as patient records that were missing vital signs or induction medications, were omitted from the analysis.

The primary outcome was PIH defined as the systemic nadir mean arterial pressure (MAP) occurring within 10 minutes following anesthetic induction. We considered absolute and percent decrease in MAP as well as post-induction MAP below 55 mm Hg, which is consistent with previous studies [10,14,16]. Both hypotension thresholds of 65 and 55 mm Hg are prevalent in practice and the literature, and the determination of the optimal MAP threshold is a matter of ongoing evaluation. Nonetheless, a stricter threshold (55 mm Hg) is rare and suggests a more impactful event less likely to have been influenced by random variation. Secondary outcomes included reactive inotrope or vasopressor use, major hemodynamic events, length of hospital stay, and 30-day mortality.

Statistical analysis

Statistical analysis was completed using Kruskal-Wallis rank sum tests, Pearson’s chi-squared tests, and logistic regression analysis. Statistical analysis was performed in Microsoft Excel (Redmond, WA, United States) and R (The R Foundation for Statistical Computing, Vienna, Austria, version 4.2.0). The Kruskal-Wallis rank sum and chi-squared tests were performed to compare continuous and discrete (categorical) patient characteristics, respectively. These characteristics are broadly categorized as Demographics, Cardiac studies, Intraoperative details, and Outcomes. Demographic characteristics include patient age, gender, BMI, ASA score, whether the surgery was listed as emergent, whether the patient had a “Do not resuscitate” in order, past medical history, and preoperative medications. Cardiac studies of interest include left ventricular ejection fraction (LVEF), left ventricular diastolic function, right ventricular function, valvular dysfunction, PASP, right atrial pressure, and WHO classification of PH. Intraoperative details include the type of surgery, whether the anesthesiologist was cardiothoracic fellowship-trained, the presence of a pre-induction arterial line, pre-induction MAP, type of airway (endotracheal tube versus laryngeal mask airway), preservation of spontaneous ventilation (that is, whether the work of breathing was performed by a mechanical ventilator), pre-emptive inotrope or vasopressor use, and primary induction agent. Lastly, our primary outcome included post-induction MAP, and secondary outcomes included reactive inotrope or vasopressor use, hospital length of stay, and 30-day mortality. Given that most of these characteristics have unknown probability distributions, a non-parametric test like the Kruskal-Wallis test is appropriate. Statistical significance was designated with two-sided p-values below 0.05 in these and all subsequent tests. Logistic regression analysis was used to estimate the odds ratios for the same patient characteristics as explanatory variables for post-induction hypotension (PIH). We performed a univariate logistic regression where the predictors were a number of variables including demographics, cardiac parameters, and intraoperative details. These included both quantitative, continuous variables (such as age), as well as categorical variables, including some binary (gender); some ordinal (ASA score), and others categorical (primary induction agent). The observed event of interest was mean arterial blood pressure nadir below 55 mm Hg, defined as a binary outcome. This analysis was performed with a two-sided 5% significance level and produced a p-value for statistical significance, odds ratios, and 95% confidence intervals for the odds ratios.

To further analyze the relationship between PH and PIH, we performed propensity score matching for our primary outcome only. We stratified patients with PASP > 70 mm Hg and those with PASP ≤ 70 mm Hg. The PASP or mean pulmonary arterial pressure (mPAP) threshold for defining PH varies in the literature. We chose a PASP of 70 mm Hg, which corresponds to the moderate to the severe range at which anesthesiologists become concerned for hemodynamic sequelae. This threshold also isolated a reasonable subsample of patients (13.5%). We assessed the causal effect of PH, for which PASP is a reasonable surrogate, where our outcome was PIH. We selected covariates as age, gender, race, height, weight, BMI, and ASA score. Covariates were limited to demographics since additional variables limited match quality, reducing the size and power of matched, comparable groups. We performed a bipartite, nearest-neighbor match. PIH, defined by blood pressure nadir within 10 minutes of anesthesia induction, was compared between matched groups.

## Results

Eight hundred ninety patients were identified and patient records were collected and stored securely. Thirty-three patients were omitted due to the aforementioned exclusion criteria. The remaining 857 patients were eligible for this study. Patient data were stratified by severity of PH according to a holistic assessment considering right ventricular function and hypertrophy. To visualize PH severity, we plot this categorization (borderline, mild, moderate, severe) against PASP, depicted in Figure 1. PASP shows reasonable separation of categories, with means ± standard deviations of 30 ± 7, 41 ± 7, 58 ± 9, and 79 ± 14, respectively.

Patient records were organized according to several personal and operative characteristics listed in Table 1. Kruskal-Wallis rank sum and chi-square tests were performed to assess for statistically significant differences in these parameters at varying levels of PH severity. Patients with more severe PH were/had older (P < 0.001), higher BMI (P = 0.002), higher ASA score (P = 0.001), higher rates of chronic kidney disease (P < 0.001), and higher rates of right or left heart failure (both P < 0.001).

**Table 1:** Demographical and clinical characteristics of the study population, stratified by the severity of pulmonary hypertension (PH). Listed P-values were obtained using Kruskal-Wallis rank sum tests or Pearson’s chi-squared tests in comparing characteristics (first column) across groups of PH severity.

	Characteristics	Borderline PH (n=142)	Mild PH (n=244)	Moderate PH (n=229)	Severe PH (n=117)	Unknown severity PH (n=125)	P-value
								Demographics	Age (years)	63 ± 14	64 ± 16	66 ± 15	68 ± 12	59 ± 15	< 0.001
Female gender	90 (63%)	149 (61%)	144 (63%)	69 (59%)	73 (58%)	0.876		
Body mass index (kg/m^2^)	31 ± 8	31 ± 8	31 ± 8	30 ± 8	35 ± 11	0.002		
ASA score						0.001		
II	6 (4%)	9 (4%)	6 (3%)	2 (2%)	3 (2%)			
III	90 (63%)	146 (60%)	116 (51%)	47 (40%)	75 (60%)			
IV	46 (32%)	88 (36%)	107 (47%)	68 (58%)	47 (37%)			
Emergent surgery	6 (4%)	9 (4%)	6 (3%)	7 (6%)	1 (1%)	0.218		
Code status for surgery						0.655		
Full code	141 (99%)	242 (99%)	224 (98%) 5 (2%)	116 (99%)	122 (98%)			
“Do not resuscitate”	1 (1%)	3 (1%)	5 (2%)	1 (1%)	3 (2%)			
Past medical history								
Hypertension	106 (75%)	189 (77%)	183 (80%)	100 (85%)	88 (70%)	0.053		
Diabetes	39 (27%)	84 (34%)	90 (39%)	56 (48%)	49 (39%)	0.012		
Chronic obstructive pulmonary disease	40 (28%)	70 (29%)	65 (28%)	41 (35%)	38 (30%)	0.718		
Chronic kidney disease	38 (27%)	79 (32%)	99 (43%)	53 (45%)	36 (29%)	<0.001		
Coronary artery disease	46 (32%)	73 (30%)	73 (32%)	31 (26%)	35 (28%)	0.793		
Carotid disease	13 (9%)	18 (7%)	17 (7%)	4 (3%)	7 (6%)	0.426		
Pulmonary embolism	17 (12%)	20 (8%)	11 (5%)	6 (5%)	9 (7%)	0.101		
Left heart failure	57 (40%)	120 (49%)	128 (56%)	73 (62%)	53 (42%)	<0.001		
Right heart failure	2 (1%)	12 (5%)	16 (7%)	25 (21%)	12 (10%)	<0.001		
Preoperative medications								
Angiotensin pathway disruptor	9 (6%)	16 (6%)	23 (10%)	11 (9%)	15 (12%)	0.315		
Beta blocker	14 (9%)	29 (12%)	32 (14%)	23 (20%)	17 (14%)	0.197		
Cardiac studies	Left ventricular ejection fraction (%)	57 ± 8	56 ± 9	54 ± 11	53 ± 10	55 ± 10	0.002
	Left ventricular diastolic function						<0.001
	Normal	35 (25%)	47 (19%)	39 (17%)	26 (22%)	23 (18%)	
	Grade I dysfunction	39 (27%)	55 (23%)	46 (20%)	27 (23%)	16 (13%)	
	Grade II dysfunction	20 (14%)	48 (20%)	60 (26%)	11 (9%)	11 (9%)	
	Grade III+ dysfunction	0 (0%)	6 (2%)	13 (6%)	11 (9%)	3 (2%)	
	Right ventricular function						<0.001
	Normal	122 (86%)	210 (86%)	174 (76%)	59 (50%)	80 (64%)	
	Mild dysfunction	14 (10%)	26 (11%)	34 (15%)	34(29%)	7 (6%)	
	Moderate dysfunction	2 (1%)	3 (1%)	10 (4%)	16 (14%)	3 (2%)	
	Severe dysfunction	0 (0%)	0 (0%)	4 (2%)	6 (5%)	2 (2%)	
	Valvular dysfunction (at least moderate severity)						
	Mitral regurgitation	7 (5%)	24 (10%)	26 (11%)	17 (15%)	3 (2%)	0.015
	Mitral stenosis	0 (0%)	5 (2%)	4 (2%)	3 (3%)	1 (1%)	0.465
	Aortic insufficiency	0 (0%)	7 (3%)	13 (6%)	4 (3%)	0 (0%)	0.009
	Aortic stenosis	3 (2%)	8 (3%)	15 (7%)	5 (4%)	3 (2%)	0.236
	Tricuspid regurgitation	4 (3%)	28 (11%)	55 24%	51 (44%)	3 (2%)	<0.001
	Tricuspid stenosis	0 (0%)	0 (0%)	0 (0%)	0 (0%)	0 (0%)	<0.001
	Pulmonic insufficiency	0 (0%)	1 (<1%)	3 (1%)	2 (2%)	0 (0%)	0.319
	Pulmonic stenosis	0 (0%)	0 (0%)	0 (0%)	0 (0%)	0 (0%)	<0.001
	Pulmonary artery systolic pressure (mm Hg)	30 ± 7	41 ± 7	58 ± 9	79 ± 14	n/a	<0.001
	Right atrial pressure (mm Hg)	4 ± 3	7 ± 5	10 ± 6	12 ± 5	6 ± 5	<0.001
	Classification of PH						<0.001
	Group 1, pulmonary arterial hypertension	8 (6%)	9 (4%)	6 (3%)	6 (5%)	7 (6%)	
	Group 2, congestive left heart disease	29 (20%)	90 (37%)	49 (21%)	35 (30%)	21 (17%)	
	Group 3, chronic hypoxic lung disease	7 (5%)	13 (5%)	26 (11%)	12 (10%)	21 (17%)	
	Group 4, chronic thromboembolic	2 (1%)	1 (<1%)	2 (1%)	3 (3%)	4 (3%)	
	Group 5, unclear/ multifactorial	43 (30%)	66 (27%)	55 (24%)	43 (37%)	19 (15%)	
	Unknown	53 (37%)	65 (27%)	59 (26%)	18 (15%)	53 (42%)	
Intraoperative details	Type of surgery						<0.001
	Cardiac	6 (4%)	9 (4%)	6 (3%)	2 (2%)	5 (4%)	
	Thoracic	5 (4%)	11 (5%)	3 (1%)	1 (1%)	3 (2%)	
	Vascular	3 (2%)	3 (1%)	9 (4%)	4 (3%)	12 (10%)	
	Ear-nose-throat/ oromaxillofacial	14 (10%)	23 (9%)	25 (11%)	8 (7%)	19 (15%)	
	Ophthalmology	1 (1%)	2 (1%)	3 (1%)	0 (0%)	0 (0%)	
	Gastroenterology	18 (13%)	35 (14%)	40 (17%)	32 (27%)	7 (6%)	
	General Surgery	11 (8%)	20 (8%)	21 (9%)	8 (7%)	10 (8%)	
	Surgical Oncology	6 (4%)	8 (3%)	7 (3%)	6 (5%)	6 (5%)	
	Obstetrics/ gynecology	10 (7%)	14 (6%)	6 (3%)	2 (2%)	5 (4%)	
	Urology	7 (5%)	23 (9%)	14 (6%)	10 (9%)	6 (5%)	
	Orthopedic	16 (11%)	34 (14%)	35 (15%)	14 (12%)	16 (13%)	
	Neurosurgery	3 (2%)	9 (4%)	3 (1%)	1 (1%)	5 (4%)	
	Trauma	6 (4%)	16 (7%)	16 (7%)	7 (6%)	2 (2%)	
	Plastic	3 (2%)	6 (2%)	8 (3%)	3 (3%)	7 (6%)	
	Transplant	3 (2%)	5 (2%)	2 (1%)	2 (2%)	7 (6%)	
	Cardiology	18 (13%)	23 (9%)	20 (9%)	10 (9%)	6 (5%)	
	Pulmonology	12 (8%)	3 (1%)	11 (5%)	7 (6%)	9 (7%)	
	Cardiothoracic anesthesiologist	50 (35%)	76 (31%)	59 (26%)	35 (30%)	44 (35%)	0.266
	Pre-induction arterial line	14 (10%)	34 (14%)	38 (17%)	29 (25%)	16 (13%)	0.005
	Pre-induction mean arterial pressure (mm Hg)	97 ± 17	98 ± 19	100 ± 20	97 ± 19	99 ± 18	0.738
	Type of airway						0.403
	Endotracheal tube	119 (84%)	212 (87%)	204 (89%)	101 (86%)	114 (91%)	
	Laryngeal mask airway	23 (16%)	32 (13%)	25 (11%)	16 (14%)	11 (9%)	
	Preservation of spontaneous ventilation	16 (11%)	15 (6%)	14 (6%)	9 (8%)	10 (8%)	0.373
	Pre-emptive inotrope or vasopressor use	20 (14%)	39 (16%)	34 (15%)	27 (23%)	24 (19%)	0.258
	Primary induction agent						0.068
	Propofol	135 (95%)	235 (96%)	212 (93%)	103 (88%)	123 (98%)	
	Etomidate	4 (3%)	3 (1%)	10 (4%)	7 (6%)	1 (1%)	
	Ketamine	1 (1%)	0 (0%)	1 (<1%)	1 (1%)	0 (0%)	
	Opioid/ benzodiazepine	0 (0%)	2 (1%)	0 (0%)	2 (2%)	0 (0%)	
	Combination	1 (1%)	4 (2%)	6 (3%)	4 (3%)	0 (0%)	
	Volatile agent	1 (1%)	0 (0%)	0 (0%)	0 (0%)	1 (1%)	
Outcomes	Post-induction decrease in mean arterial pressure (mm Hg)	31 ± 17	33 ± 19	32 ± 19	31 ± 21	31 ± 23	0.862
	Post-induction % decrease in mean arterial pressure	32 ± 15	33 ± 17	31 ± 16	31 ± 19	30 ± 20	0.805
	Post-induction mean arterial pressure <55 mm Hg	39 (27%)	76 (31%)	56 (24%)	33 (28%)	31 (25%)	0.495
	Reactive inotrope or vasopressor use	60 (42%)	103 (42%)	100 (44%)	54 (46%)	49 (39%)	0.863
	Major post-induction hemodynamic events	0 (0%)	1 (<1%)	3 (1%)	0 (0%)	0 (0%)	0.256
	Post-operative hospital length of stay	5 ± 15	5 ± 9	5 ± 9	9 ± 18	5 ± 7	0.009
	30-day mortality	1 (1%)	2 (1%)	5 (2%)	6 (5%)	3 (2%)	0.061

A number of cardiac parameters also correlated with severity of PH (Table 1). These include left ventricular diastolic dysfunction (P < 0.001); right ventricular dysfunction (P < 0.001); valvular dysfunction of at least moderate severity, including tricuspid regurgitation (P < 0.001) and mitral regurgitation (P = 0.015); PASP (P < 0.001); right atrial pressure (P = 0.001); and WHO classification of PH (P = 0.001), as defined by chart review for these diagnoses including the respective ICD codes and on echocardiogram reports. Pre-induction arterial lines were also more prevalent with increasing severity of PH (P = 0.005). Lastly, patients with more severe PH had a longer post-operative length of stay in the hospital (P = 0.009).

We next performed univariate logistic regression analysis to identify which of these characteristics are significant predictors of PIH defined as a MAP below 55 mm Hg, with results depicted in Table 2. According to our definition of statistical significance (P < 0.05), none of our tested parameters significantly predicted PIH. Right heart failure was the strongest trend with PIH in patients with PH (P = 0.052), with odds ratio 1.116 and 95% confidence interval (0.999, 1.127). We also found that diabetes was associated with decreased rates of PIH, with odds ratio 0.919 (P = 0.007). We lastly found that patients who maintained spontaneous ventilation were also less likely to have PIH (odds ratio 0.925 with P = 0.012).

**Table 2:** Univariate logistic regression analysis of predictors of a post-induction mean arterial pressure of < 55 mm Hg.

	Predictors	Odds ratio (95% Conf int)	P-value
Demographics	Age (years)	1.001 (0.999, 1.003)	0.390
	Female gender	1.038 (0.977, 1.104)	0.337
	Body mass index (kg/m^2^)	1.000 (0.996, 1.003)	0.917
	ASA score	0.977 (0.925, 1.032)	0.411
	Emergent surgery	1.041 (0.883, 1.227)	0.634
	Code status for surgery	1.018 (0.902, 1.151)	0.769
	Past medical history		
	Hypertension	0.953 (0.887, 1.024)	0.190
	Diabetes	0.919 (0.864, 0.977)	0.007
	Chronic obstructive pulmonary disease	0.986 (0.924, 1.053)	0.679
	Chronic kidney disease	0.949 (0.891, 1.010)	0.097
	Coronary artery disease	0.948 (0.888, 1.012)	0.109
	Carotid disease	1.005 (0.892, 1.131)	0.941
	Pulmonary embolism	0.998 (0.89, 1.119)	0.973
	Left heart failure	0.956 (0.901, 1.015)	0.142
	Right heart failure	1.116 (0.999, 1.247)	0.052
	Preoperative medications		
	Angiotensin pathway disruptor	1.003 (0.901, 1.116)	0.963
	Beta blocker	1.031 (0.944, 1.126)	0.494
Cardiac studies	Left ventricular ejection fraction (%)	1.003 (0.999, 1.006)	0.132
	Left ventricular diastolic function	0.988 (0.965, 1.011)	0.301
	Right ventricular function	0.998 (0.948, 1.051)	0.941
	Valvular dysfunction (at least moderate severity)		
	Mitral regurgitation	1.013 (0.912, 1.124)	0.815
	Mitral stenosis	0.957 (0.749, 1.222)	0.723
	Aortic insufficiency	0.857 (0.715, 1.028)	0.096
	Aortic stenosis	0.903 (0.775, 1.052)	0.192
	Tricuspid regurgitation	1.031 (0.951, 1.119)	0.456
	Tricuspid stenosis	NA	NA
	Pulmonic insufficiency	0.897 (0.627, 1.284)	0.553
	Pulmonic stenosis	NA	NA
	Pulmonary artery systolic pressure (mm Hg)	1.000 (0.998, 1.002)	0.863
	Right atrial pressure (mm Hg)	1.001 (0.995, 1.007)	0.699
	Classification of PH	1.002 (0.985, 1.02)	0.786
Intraoperative details	Type of surgery	1.003 (0.996, 1.01)	0.364
	Cardiothoracic anesthesiologist	0.977 (0.916, 1.043)	0.487
	Pre-induction arterial line	1.012 (0.932, 1.098)	0.780
	Pre-induction mean arterial pressure (mm Hg)	0.992 (0.991, 0.994)	<0.001
	Type of airway (endotracheal tube vs laryngeal mask)	0.978 (0.893, 1.07)	0.178
	Preservation of spontaneous ventilation	0.925 (0.826, 1.036)	0.012
	Pre-emptive inotrope or vasopressor use	1.107 (1.023, 1.199)	0.553
	Primary induction agent	0.986 (0.941, 1.033)	0.178
	Propofol	1.000 (0.999, 1.000)	0.518
	Etomidate	1.016 (0.991, 1.041)	0.217
	Ketamine	1.001 (0.997, 1.006)	0.600

**Figure 2:**
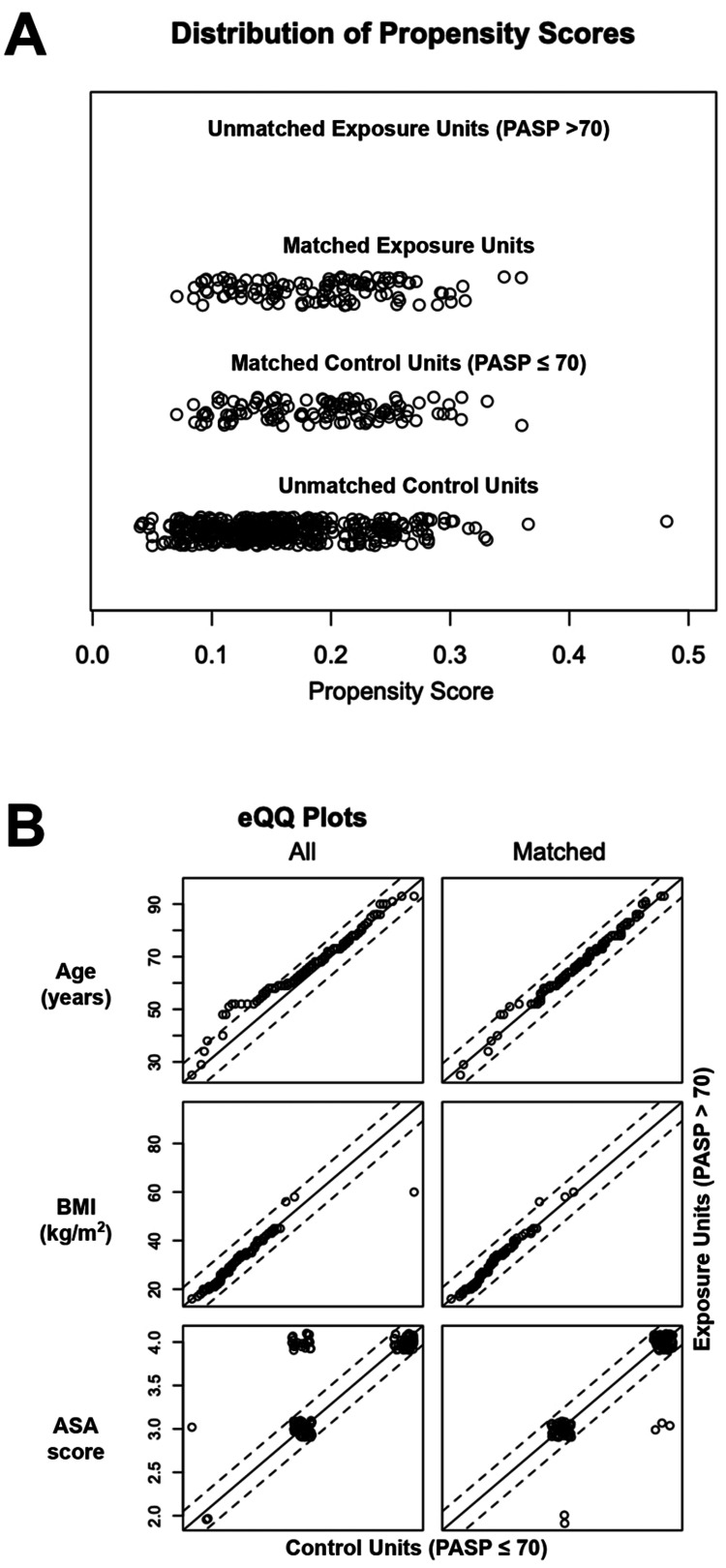
Propensity score matching demonstrates no significant difference (P = 0.29) between patients with high and low PASP (A) Matching across several covariates produced 116 patients in each exposure and control groups with similar propensity scores. (B) The empirical quantile-quantile (eQQ) plots show the successful elimination of outliers during the matching process.

We next performed propensity score matching to further understand this relationship between PH and PIH. Of the 857 patients eligible for this study, 116 had PASP > 70, which provides a surrogate for the severity of PH (Figure 1). From this histogram, it is clear that the gradations of PH have minimal overlap when ordered according to PASP. The regions of overlap might correspond to samples where PASP does not correlate with PH, such as in the setting of tricuspid regurgitation or when CVP cannot be accurately estimated using IVC diameter. The graded severities of PH, as depicted in Figure 1, are considered exposures. These 116 patients were matched to 116 controls with PASP ≤ 70 mm Hg according to age, gender, race, BMI, and ASA score. The resultant match was successful, as depicted in Figure 2. The propensity scores of matched and unmatched exposures and controls are depicted in Figure 2A, showing a near-equivalent match. To further depict goodness-of-match, we show empirical quantile-quantile (eQQ) plots of age, BMI, and ASA score in Figure 2B, which shows the elimination of outliers in these covariates. When comparing matched control and exposure groups, the blood pressure nadir within ten minutes of anesthesia induction was not significantly different (P = 0.29), suggesting that PH, defined by PASP > 70, does not predict PIH.

## Discussion

The aim of this study was to identify predictors of PIH in patients undergoing surgery with a diagnosis of PH, yet no strong, definitive predictors were found. PH is a serious condition well known to burden cardiac function and the blood pressure perturbations that occur with the induction of general anesthesia incur additional sudden and dramatic hemodynamic stress. To the authors’ knowledge at the time this manuscript was written, this is the first attempt to describe the extent and clinical impact of PIH in this particularly vulnerable population. Our analysis revealed significant associations between age, BMI, ASA class, and renal/cardiac comorbidities with PH severity, yet none of our tested parameters were significantly predictive for PIH in patients with PH.

We first stratified patients according to the severity of the PH and found characteristics strongly associated with these groups. We found that renal and cardiac comorbidities (chronic kidney disease and right/left heart failure) are associated with PH. This is intuitive given the strong correlations and pathophysiologic sequence between left heart failure, PH, and right heart failure [17,18]. The structural consequences of chronic hypertension on arteries of the heart and kidney are well established [19,20]. These structural changes, mainly medial thickening and increased stiffness, can also contribute to heart failure [21]. PH, kidney failure, and heart failure are all sequela of chronic systemic hypertension.

We then identified predictors of PIH in our patient population with PH. Right heart failure showed the most significant trend in patients with PIH. This was expected given the increased right ventricular afterload and resultant reduced left ventricular preload [17]. We found that patients who were maintained spontaneously ventilating were less likely to have PIH. This may have been due to the minimization of right ventricular afterload afforded by avoiding positive pressure ventilation; however, this result may also have been confounded by the depth of anesthesia. Patients who received lower doses of anesthesia medications, carefully titrated to preserve spontaneous ventilation, may not have experienced as much hypotension as a result. Similarly, patients for whom the preservation of spontaneous ventilation was not attempted may have received higher doses of anesthesia medications and may have been more likely to experience PIH.

We also found that diabetes has a weak association with decreased rates of PIH. Although the mechanism for this observation remains unclear, it is possibly related to the dysfunction of the autonomic nervous system. Another potential mechanism is the protective effect of diabetic medications, which is incompletely understood. It is important to note that although this finding was statistically significant (P = 0.007) and a substantial fraction of the study population (38%) had diabetes, the effect size was small (odds ratio 0.919). This association is therefore unlikely to be a statistical aberrancy. Nonetheless, further research is required before concluding that diabetes might have a protective effect against PIH in patients with PH. 

We surprisingly found that some predictors of PIH in the broader population as determined in prior studies [10], namely age, ASA score, and propofol as an induction agent, were not significant predictors in the PH population here. One possible explanation is that most patients in our cohort (approximately 95%) received propofol as a primary induction agent and leaving a small comparison group (approximately 5%). Another possible explanation is that patients with PH already have altered hemodynamics such that these parameters do not further predispose them to PIH. Contrastingly, in the general population that does not have clinically significant PH, these predictors (age, ASA score, and propofol) might have stronger hemodynamic consequences. It is also surprising that the classification of PH based on severity did not strongly predict PIH, and that propensity score matching revealed no significant difference in PIH. Given the abundant anesthetic considerations for this patient population, including specific recommendations to prevent hypotension [8,22,23], we expected that the severity of PH would have been strongly associated with PIH. The degree of PH was not a significant predictor of PIH, with an odds ratio of 1.002 (P = 0.786). Thus, unfortunately, the role of PH in the consideration of PIH remains unclear. It is possible this condition is not as consequential in the peri-induction phase as previously thought. However, our singular study and its retrospective nature should prompt caution in the interpretation of these results. It is very likely that the highest-risk patients were not included in this study. The incidence of case cancellations and/or avoidance of general anesthesia based on the decision that a patient had unacceptably increased perioperative risk was not captured in our analysis. It is important to emphasize that, in spite of our preliminary findings, the established anesthetic considerations and precautionary measures for patients with PH remain strongly justified to prevent morbidity and mortality [11,12,23].

This study has a few key strengths that differentiate it from previous studies. No study thus far has considered predictors of PIH specific to patients with PH and discretized by the severity of PH. This study analyzes a moderate-sized dataset of patients with wide-ranging comorbidities undergoing a broad spectrum of procedures. We also used a variety of statistical analyses-Pearson’s chi-squared test, logistic regression, and propensity score matching-to reinforce our assessment of the relationship between PH and PIH. Such contributions improve our understanding of how specific conditions like PH contribute to anesthetic care.

In addition to some of the aforementioned limitations, there are others worth considering when interpreting these results. We performed a single analysis of a moderate sample size from one institution, so these results might not be generalizable to other regions and populations. This study was on a heterogeneous population with varying etiologies for PH undergoing varying types of surgery, not controlled by clinician experience or type of anesthetic induction. Moreover, it is possible that patients in this study with more severe PH implicitly and explicitly received a higher level of care. Indeed, the patients with increased severity PH had a higher frequency of arterial lines, and patients with spontaneous respiration had lower severities of PH. It is possible that these and other unaccounted, implicit factors confound our results and eliminate weaker causes of PIH identified in previous studies. Further, it is possible that our selection criteria produced an inherently biased population for the study. Inherent to retrospective studies are confounding factors that are unaccounted for based on the study design. Although our study assessed predictors of PIH and identified associations with PH, there may be other parameters that were not considered or possible. For example, preoperative comprehensive cardiac catheterization data, though the gold standard in characterizing the severity and nature of PH, were too sporadically available to include in our analysis. That said, PASP estimated by echocardiography is more widely available and has been shown to correlate well with catheterization measurements. Although PASP correlated well with the severity of PH, there was a clear overlap between categories, and PASP is not the perfect surrogate for PH in our propensity score analysis. A subgroup analysis with cases of confirmed PH with right heart catheterization data might produce positive results. Many of these limitations can also be resolved by repeating similar studies and observing prospective cohorts undergo a similar assessment.

## Conclusions

In this study, we analyzed predictors of PIH for patients with PH undergoing surgery with general anesthesia using the Kruskal-Wallis rank sum test, Pearson’s chi-squared test, logistic regression analysis, and propensity score matching. We found strong correlations between PH severity and patient age, BMI, ASA class, as well as renal and cardiac comorbidities; however, none of our tested parameters shows a statistically significant prediction of PIH in patients with PH. Right heart failure had the strongest trend with PIH, whereas diabetes and maintenance of spontaneous ventilation were significantly associated with decreased rates of PIH. These results challenge the longstanding hesitations for PIH in the anesthetic care of patients with PH. However, any clinical implications of this work should await prospective studies and repeat analyses.

## References

[1] Simonneau G, Robbins IM, Beghetti M (2009). Updated clinical classification of pulmonary hypertension. J Am Coll Cardiol.

[2] Hoeper MM, Humbert M, Souza R (2016). A global view of pulmonary hypertension.. Lancet Respirat Med.

[3] Moreira EM, Gall H, Leening MJ (2015). Prevalence of pulmonary hypertension in the general population: the Rotterdam study. PLoS One.

[4] Naeije R, Manes A (2014). The right ventricle in pulmonary arterial hypertension. Eur Respir Rev.

[5] Rosenkranz S, Howard LS, Gomberg-Maitland M, Hoeper MM (2020). Systemic consequences of pulmonary hypertension and right-sided heart failure. Circulation.

[6] Smilowitz NR, Armanious A, Bangalore S, Ramakrishna H, Berger JS (2019). Cardiovascular outcomes of patients with pulmonary hypertension undergoing noncardiac surgery. Am J Cardiol.

[7] Lai HC, Lai HC, Wang KY, Lee WL, Ting CT, Liu TJ (2007). Severe pulmonary hypertension complicates postoperative outcome of non-cardiac surgery. Br J Anaesth.

[8] McGlothlin D, Ivascu N, Heerdt PM (2012). Anesthesia and pulmonary hypertension. Prog Cardiovasc Dis.

[9] Südfeld S, Brechnitz S, Wagner JY, Reese PC, Pinnschmidt HO, Reuter DA, Saugel B (2017). Post-induction hypotension and early intraoperative hypotension associated with general anaesthesia. Br J Anaesth.

[10] Reich DL, Hossain S, Krol M, Baez B, Patel P, Bernstein A, Bodian CA (2005). Predictors of hypotension after induction of general anesthesia. Anesth Analg.

[11] Walsh M, Devereaux PJ, Garg AX (2013). Relationship between intraoperative mean arterial pressure and clinical outcomes after noncardiac surgery: toward an empirical definition of hypotension. Anesthesiology.

[12] Bijker JB, Persoon S, Peelen LM, Moons KG, Kalkman CJ, Kappelle LJ, van Klei WA (2012). Intraoperative hypotension and perioperative ischemic stroke after general surgery: a nested case-control study. Anesthesiology.

[13] Chen B, Pang QY, An R, Liu HL (2021). A systematic review of risk factors for postinduction hypotension in surgical patients undergoing general anesthesia. Eur Rev Med Pharmacol Sci.

[14] Kendale S, Kulkarni P, Rosenberg AD, Wang J (2018). Supervised machine-learning predictive analytics for prediction of postinduction hypotension. Anesthesiology.

[15] Kang AR, Lee J, Jung W, Lee M, Park SY, Woo J, Kim SH (2020). Development of a prediction model for hypotension after induction of anesthesia using machine learning. PLoS One.

[16] Vos JJ, Scheeren TW (2019). Intraoperative hypotension and its prediction. Indian J Anaesth.

[17] Bogaard HJ, Abe K, Vonk Noordegraaf A, Voelkel NF (2009). The right ventricle under pressure: cellular and molecular mechanisms of right-heart failure in pulmonary hypertension. Chest.

[18] Alcantara CA, Chandra A, Morvey D, von Schwarz ER (2018). Acute right heart failure. Right Heart Pathology.

[19] Folkow B (1993). Early structural changes in hypertension: pathophysiology and clinical consequences. J Cardiovasc Pharmacol.

[20] Schiffrin EL (1992). Reactivity of small blood vessels in hypertension: relation with structural changes. State of the art lecture. Hypertension.

[21] Slivnick J, Lampert BC (2019). Hypertension and heart failure. Heart Fail Clin.

[22] Pritts CD, Pearl RG (2010). Anesthesia for patients with pulmonary hypertension. Curr Opin Anaesthesiol.

[23] Zafirova Z, Rubin LJ, Hines R, Yeon SB (2022). Anesthesia for patients with pulmonary hypertension or right heart failure. https://www.uptodate.com/contents/anesthesia-for-noncardiac-surgery-in-patients-with-pulmonary-hypertension-or-right-heart-failure.

